# Angiotensin-(1–7) infusion in COVID-19 patients admitted to the ICU: a seamless phase 1–2 randomized clinical trial

**DOI:** 10.1186/s13613-024-01369-0

**Published:** 2024-09-04

**Authors:** Ana Luiza Valle Martins, Filippo Annoni, Filipe Alex da Silva, Lucas Bolais-Ramos, Gisele Capanema de Oliveira, Renata Cunha Ribeiro, Mirella Monique Lana Diniz, Thuanny Granato Fonseca Silva, Beatriz Dias Pinheiro, Natália Abdo Rodrigues, Alana Helen dos Santos Matos, Daisy Motta-Santos, Maria José Campagnole-Santos, Thiago Verano-Braga, Fabio Silvio Taccone, Robson Augusto Souza Santos

**Affiliations:** 1 National Institute of Science and Technology in Nanobiopharmaceutics (INCT-Nanobiofar), Laboratory of Hypertension, Institute of Biological Sciences, Department of Physiology and Biophysics, Federal University of Minas Gerais, Belo Horizonte, Minas Gerais Av. Antonio Carlos, 6627-ICB-UFMG, Belo Horizonte, 31270-901 Brazil; 2 Department of Intensive Care Erasme Hospital, University Hospital of Brussels (HUB), Lennik Road 808, 1070 Brussels, Belgium

**Keywords:** Angiotensin, ARDS, Coronavirus, Renin angiotensin system, RAS, COVID-19

## Abstract

**Background:**

The coronavirus-related disease (COVID-19) is mainly characterized by a respiratory involvement. The renin-angiotensin system (RAS) has a relevant role in the pathogenesis of COVID-19, as the virus enters host’s cells via the angiotensin-converting enzyme 2 (ACE2).

**Methods:**

This investigator-initiated, seamless phase 1–2 randomized clinical trial was conceived to test the safety and efficacy of continuous short-term (up to 7 days) intravenous administration of Angiotensin-(1–7) in COVID-19 patients admitted to two intensive care units (ICU). In addition to standard of care, intravenous administration of Angiotensin-(1–7) was started at 5 mcg/Kg day and increased to 10 mcg/Kg day after 24 h (Phase 1; open label trial) or given at 10 mcg/Kg day and continued for a maximum of 7 days or until ICU discharge (Phase 2; double-blind randomized controlled trial). The rate of serious adverse events (SAEs) served as the primary outcome of the study for Phase 1, and the number of oxygen free days (OFDs) by day 28 for Phase 2.

**Results:**

Between August 2020 and July 2021, when the study was prematurely stopped due to low recruitment rate, 28 patients were included in Phase 1 and 79 patients in Phase 2. Of those, 78 were included in the intention to treat analysis, and the primary outcome was available for 77 patients. During Phase 1, one SAE (i.e., bradycardia) was considered possibly related to the infusion, justifying its discontinuation. In Phase 2, OFDs did not differ between groups (median 19 [0–21] vs. 14 [0–18] days; p = 0.15). When patients from both phases were analyzed in a pooled intention to treat approach (Phase 1–2 trial), OFDs were significantly higher in treated patients, when compared to controls (19 [0–21] vs. 14 [0–18] days; absolute difference −5 days, 95% CI [0–7] p = 0.04).

**Conclusions:**

The main findings of our study indicate that continuous intravenous infusion of Angiotensin-(1–7) at 10 mcg/Kg day in COVID-19 patients admitted to the ICU with severe pneumonia is safe. In Phase II intention to treat analysis, there was no significant difference in OFD between groups.

*Trial Registration* ClinicalTrials.gov Identifier: NCT04633772—Registro Brasileiro de Ensaios Clínicos, UTN number: U1111-1255-7167.

**Supplementary Information:**

The online version contains supplementary material available at 10.1186/s13613-024-01369-0.

## Introduction

The pandemic caused by the novel Coronavirus (SARS-CoV-2) has been responsible for a high death toll worldwide, reaching over 7 million by January 2024 [1]. The Coronavirus disease (COVID-19) can manifest with a large spectrum or respiratory involvement, ranging from mild and self-limiting disease to fast progressive bilateral pneumonia, eventually leading to death [2–5]. Up to date, only a few immuno-modulatory agents (i.e., corticosteroids, anti-IL6 agents) have been recommended in COVID-19 patients hospitalized in intensive care units (ICUs) [6–8]. However, there is no consistent data on outcomes with anti-viral drugs, such as remdesivir, for severely ill COVID-19 patients.

The pathophysiology of the disease remains complex, and the development of new, cost-effective medical treatments is crucial to mitigate the impact of COVID-19 and its variants, while also laying the foundation for potential treatments during future epidemics. Specifically, the renin-angiotensin system (RAS) plays a pivotal role, with the angiotensin-converting enzyme 2 (ACE 2) protein serving as the cellular binding site for the spike proteins of SARS-CoV-2. This interaction facilitates viral entry into the cell, subsequently enabling viral replication [9].

Angiotensinogen, a protein primarily synthesized in the liver, undergoes transformation into Angiotensin I (Ang I) through the action of renin, followed by cleavage into Angiotensin II (Ang II) by ACE, a dipeptidyl carboxypeptidase. Subsequently, Ang II can initiate various responses in multiple tissues by binding to its specific receptors, known as AT1 and AT2. Notably, both Ang I and Ang II can be further processed by endopeptidases and ACE2 to yield Angiotensin 1–7 (Ang-(1–7), which binds to a specific receptor, MasR, initiating a cascade of biological responses [10, 11]. It is worth highlighting that the ACE2/Ang-(1–7)/MasR pathway counterbalances the effects of the ACE/Ang II/AT1 pathway, exerting a significant modulatory influence on various biological processes. These include the regulation of inflammatory responses, tissue fibrosis, blood pressure, renal function, angiogenesis, as well as endocrine and hormonal functions [10, 11].

Considerable preclinical and experimental evidence suggests that the activation of the pulmonary-based RAS plays a pivotal role in the pathophysiology of pulmonary inflammation [12–17]. Conversely, the ACE2/Ang-(1–7)/Mas receptor pathway has demonstrated anti-inflammatory properties in various pulmonary disorders [12–21]. In patients with COVID-19, plasmatic Ang-(1–7) levels have shown a slight increase, with a reduction in Ang II levels when compared to healthy individuals [22, 23]. Nevertheless, the specific evaluation of lung RAS dysregulation and relative Ang-(1–7) insufficiency in these patients has not been conducted.

Current evidence suggests that discontinuation of both ACE inhibitors (ACEi) and angiotensin II receptor blockers (ARBs) is not warranted in non-critically ill COVID-19 patients [24, 25]. However, the initiation of these medications has been associated with adverse outcomes in critically ill patients [26]. To date, only a small series of patients and a randomized controlled trial have investigated the potential benefits of intravenous Ang-(1–7) in COVID-19 patients, yielding neutral results [27, 28]. Notably, both trials employed a therapeutic approach involving a single daily 3-h infusion of high-dose Ang-(1–7), potentially eliciting distinct biological responses due to the compound's short half-life and relative affinity for other receptors within the RAS [29, 30].

The aim of this seamless Phase 1 and 2 study was therefore to assess the safety of a dose-escalating short-term continuous intravenous administration of Ang-(1–7) in COVID-19 patients admitted in the ICU, and to investigate its efficacy on pulmonary function.

## Methods

### Study design and population

This investigator-initiated trial was conducted in a sequential seamless Phase 1–2 design. The trial comprised two distinct phases: Phase 1 was an open-label, dose-escalating clinical trial, followed by Phase 2, which was a randomized, controlled, double-blind clinical trial. The study was carried out at two ICUs, namely Mater Dei Hospital and Eduardo de Menezes Hospital, located in Belo Horizonte, Minas Gerais, Brazil.

Eligible participants were adults aged between 18 and 80 years, presenting with confirmed or highly suspected COVID-19 (positive contact or suggestive image) and requiring ICU admission with clinical signs of pneumonia plus one of the following criteria: respiratory rate greater than 30/min; signs of respiratory effort, SatO2 < 90% in room air). Exclusion criteria included patients diagnosed with cancer, those requiring high-dose vasopressors (i.e., norepinephrine > 0.5 mcg/kg min), individuals with chronic immune system depression (i.e., chronic intake of immunosuppressive drugs, inherited known immunological disorders, or known HIV infection), patients with active care limitations, those with primary respiratory failure caused by cardiac issues, with idiopathic lung fibrosis, individuals undergoing chronic dialysis, patients with acute on chronic liver failure decompensated liver cirrhosis, individuals on chronic home oxygen therapy, pregnant women or those participating in any other interventional trial.

The inclusion screening and randomization of consecutive patients were contingent on the availability of research personnel at the time of admission. Ethical approval for the study protocol was obtained from the Ethics Committees of the participating centers. Written informed consent was acquired from either a legal representative or the patient, as appropriate, depending on the circumstances. The study adhered to CONSORT recommendations [31], with the checklist available in Appendix 1.

### Randomization and blinding

Patients' eligibility for the Phase 2 trials was assessed at ICU admission. Study personnel recorded patients’ characteristics in a centralized electronic randomization system (REDCap). Patient-level randomization was carried out using permuted blocks of varying sizes, with stratification based on the presence of hypertension, diabetes, obesity, the use of ACEi/ARBs and age over 60 years automatically performed by system (REDCap).

The outcome of the randomization process was provided to the hospital pharmacist in the form of a batch number, which was then used to prepare the drug for infusion. The specific infusion details were known only to the manufacturer responsible for supplying the formulation for intravenous infusion of the investigational product. Consequently, patients, treating clinicians, paramedics, and study personnel remained blinded to the nature of the treatment throughout the Phase 2 study. Following the database lock, the manufacturer disclosed the details of the administered batch to the principal investigator.

### Study intervention

Patients meeting the inclusion criteria received continuous intravenous administration of Ang-(1–7)—provided as a peptide by BCN Peptides, Barcelona, Spain and prepared for intravenous infusion by Citopharma Manipulação de Medicamentos Especiais LTDA, Belo Horizonte, Minas Gerais, Brazil. The peptide was administered through a dedicated intravenous line, either central or peripheral, after dilution in a NaCl 0.9% solution.

During Phase 1, patients initially received a dose of 5 mcg/kg day, which was increased to 10 mcg/kg day after 24 h. This treatment regimen was continued for a maximum of 7 days or until clinical improvement resulting in ICU discharge or death, whichever occurred first. In Phase 2, treatment commenced with a dose of 10 mcg/kg day, while the infusion duration remained the same. Decisions regarding other interventions, such as the use of noninvasive ventilation or mechanical ventilation, renal replacement therapy, vasopressors, or extracorporeal membrane oxygenation, were left to the discretion of the attending physician, as were determinations regarding the withdrawal of life-sustaining therapies. The trial protocol did not prescribe or influence the use of immunomodulatory therapies or other specific therapeutic regimens, including anticoagulants.

### Study outcomes

The primary outcome in Phase 1 was the occurrence of Severe Adverse Events (SAEs), which were recorded after the initiation of therapy and throughout the drug administration period. Any SAEs were promptly reported to the Data Safety Monitoring Committee (DSMC) for evaluation. These SAEs were categorized as related or unrelated to the study medication and were defined in accordance with the criteria outlined in Appendix 2. In particular, episodes of severe hypotension requiring adjunctive treatment, acute myocardial infarction or stroke and massive pulmonary embolism and TVP were recorded during Phase 1 regardless of their incidence.

In Phase 2, the primary outcome was the number of oxygen-free days (OFDs) by day 28. OFDs represent a composite outcome that considers both mortality and the duration of oxygen support within a predefined timeframe, with censoring at 28 days. Secondary outcomes encompassed the following: (a) length of ICU stay; (b) ICU mortality; (c) length of stay in the hospital; (d) ventilator-free days by day 28, signifying the number of days patients were liberated from mechanical ventilation from inclusion until day 28; (e) requirement for vasopressors; (g) circulating levels of Ang I, Ang II, Ang-(1-5), and Ang-(1-7), which were measured at baseline (T0), 3 h (T1), 24 h (T2), and 72 h (T3) after the initiation of infusion, as well as on day 7. Quantification of these RAS peptides was performed using a mass spectrometry tandem liquid chromatography approach, as previously documented [23].

### Statistical analysis

Given the absence of prior data and the exploratory nature of the trial, a convenient sample size of 30 patients was deemed sufficient for evaluating the safety of the investigational drug (Phase 1). Subsequently, an amendment was made to the protocol to reduce the required number of patients (Amendment number 4,277,977, CAAE 34080720.0.1001.5149). For Phase 2, a sample size of 100 patients was considered appropriate. This size was deemed adequate to provide substantial data for planning a larger Phase 3 clinical trial in the event of demonstrated clinical efficacy.

Descriptive statistics were computed for all variables in the study. Categorical data were presented as counts and percentages, while continuous data were expressed as either mean (± standard deviation) or median [25th–75th percentiles], depending on the distribution of each variable, which was assessed using the Kolmogorov–Smirnov test. Unpaired t-tests or Mann-Whitney tests were applied as appropriate for continuous variables, while Chi-square or Fisher’s exact tests were used for categorical variables. To compare quantitative variables across different time points, a linear mixed model fitted for restricted maximum likelihood estimation (REML), with Gasser-Greenhouse correction, was employed. Multiple comparisons were adjusted using the Sidak method. For the primary outcome, a secondary analysis considered subgroups based on age, sex at birth, presence of obesity and previous treatment with ACE inhibitors or sartans. The significance level set for analyses was 0.05.

Both an intention-to-treat and per-protocol analysis, considering only patient who adhered to the infusion protocol as planned, were conducted to assess the primary outcome of Phase 2. An intention-to-treat approach was utilized for the exploratory analysis of pooled data from both phases. IBM SPSS Statistics software, version 25, and GraphPad Prism (version 9.3.1 for Macintosh, GraphPad Software, La Jolla, CA, US) were employed for statistical analyses.

## Results

### Study population

Between August and December 2020, a total of 29 patients were enrolled in Phase 1; however, one patient was subsequently excluded after new information revealed a prior diagnosis of active cancer before drug administration, resulting in a final analysis cohort of 28 patients. The mean age of this study cohort was 55.8 ± 12.0 years, with an average body mass index (BMI) of 31.1 (± 7.3) Kg/m^2^. Prior to admission, 11 patients (39.2%) were receiving chronic therapy with ACEi/ARBs. The median length of ICU was 5 [4–7] days, and 6 out of 28 (21.4%) patients did not survive.

**Fig. 1:**
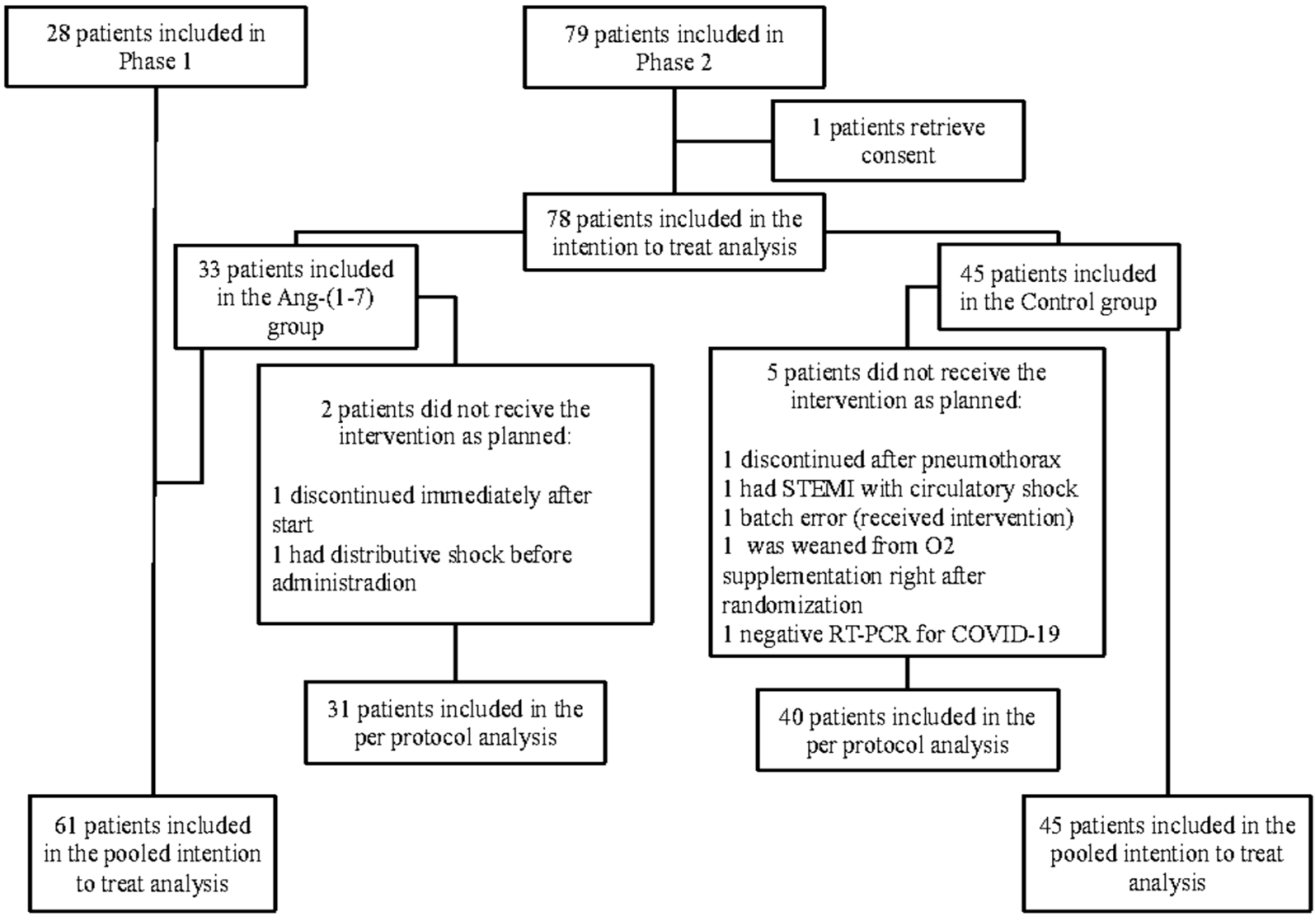
Flowchart of the study

Between December 2020 and July 2021, a total of 79 patients were randomized for Phase 2, with one patient subsequently withdrawing consent, resulting in 78 patients available for the primary intention-to-treat analysis. In July 2021, due to a low recruitment rate and funding difficulties, enrollment in the study was halted. The inclusion process is illustrated in Figure 1. Notably, patients in the intervention group had a higher BMI and a lower pH at inclusion, when compared to the control group. During Phase 2, 16 out of 79 patients (20.2%) died. Detailed participant characteristics for both phases are outlined in Table 1. All patients were included within the first 48 h from ICU admission. From all phase II patients, 7 received recombinant humanized anti-interleukin-6 receptor (IL-6R) monoclonal antibody Tocilizumab (5 in the intervention group, and 2 in the placebo group), no other IL-6 blockers were used.

**Table 1:** characteristics of the study population

	Phase 1	Phase 2	
		Ang-(1–7)	Control
Demographics			
Age at inclusion, years	55.9 (12.5)	51.9 (13.2)	56.5 (12.3)
Body Mass Index, Kg/m^2^	31.1 (7.3)	33.4 (6.8)	30.3 (5.1)
Male, n	22 (78.6)	22 (66.6)	28 (60.8)
Comorbidities			
Arterial hypertension, n	13 (46.4)	11 (35.4)	16 (38)
ACEi, n	3 (10.7)	1 (3.2)	1 (2.2)
ARBs, n	8 (28.6)	5 (16.1)	9 (20.4)
Chronic heart disease, n	1 (3.6)	2 (6.4)	0
Diabetes, n	10 (35.7)	5 (17.2)	15 (35.7)
Chronic neurological disorder, n	1 (3.6)	0	1 (2.2)
Dementia, n	1 (3.6)	0	1 (2.2)
Obesity (BMI > 30), n	11 (39.2)	14 (45.1)	16 (36.6)
Chronic pulmonary disease, n	1 (3.6)	0	0
Asthma, n	2 (7.1)	3 (9.6)	1 (2.2)
Active smoking, n	2 (7.1)	0	2 (4.5)
Chronic liver disease, n	0	1 (3.2)	0
Chronic kidney disease, n	1 (3.6)	1 (3.2)	0
Parameters at inclusion			
Temperature, °C	36.7 (0.8)	36.8 (0.4)	36.8 (0.9)
Heart rate, beats/min	94.2 (17.3)	82 (9.5)	81 (10.8)
Respiratory rate, /min	23.8 (5.6)	24.5 (18–28)	22 (18–27)
Systolic blood pressure, mmHg	137.2 (19.1)	120 (114.8–130)	120 (110–141)
Diastolic blood pressure, mmHg	79.0 (12.7)	75.5 (68.5–80)	75 (70–80)
SaO_2_, %	94.1 (3.2)	94 (92–96)	93 (90–95)
FiO_2_, %	52.0 (27.5)	50 (33–80)	70 (37–90)
Mechanical ventilation, n	9 (32.1%)	3 (9.6)	8 (18.1)
Non-invasive ventilation, n	2 (7.1%)	4 (12.9)	2 (4.5)
Corticoids, n	28 (100%)	28 (93.3)	42 (100)
Vasopressor support, n	8 (28.5%)	3 (10)	7 (15)
NMBA, n	8 (28.5%)	3 (9.6)	8 (17.7)
First available			
pH	7.44 (7.41–7.46)	7.42 (7.40–7.45)	7.44 (7.42–7.47)
Lactate, mmol/L	1.4 (1.2–1.8)	1.3 (1.1–1.8)	1.3 (1.1–1.7)
PaO_2_/FiO_2_	173 (111–231)	104.1 (81.6–168)	115 (90–166)
PaCO_2_, mmHg	38.3 (33–46)	39 (35–42)	37 (33–42)
CRP, mg/dL	153(29–415)	166 (105–218)	125 (87–200)
Hemoglobin, g/dL	13.3 (11.5–14)	13.4 (11.9–14)	12.8 (11.9–13.9)
APACHE II score	6 (2–15)	9 (6–12)	9 (7–10)

Data are presented as mean (± standard deviation), count (%) or median value (interquartile range)

*ACEi* angiotensin converting enzyme inhibitors, *ARBs* angiotensin receptor blockers, *BMI* body mass index, *SaO*_*2*_ arterial oxygen saturation, *FiO*_*2*_ inspired fraction of oxygen, *NMBA* neuromuscular blocking agents, *PaO*_*2*_ arterial partial oxygen pressure, *PaCO*_*2*_ arterial partial carbon dioxide pressure, *CRP* C-reactive protein, *APACHE II* acute physiology and chronic health evaluation score II

#### Primary outcomes

In Phase 1, the median duration of Ang-(1–7) infusion was 4 ± 2 days, and dose escalation was possible for all patients except one. In two patients (7.1%), the infusion was temporarily discontinued on multiple occasions due to technical issues unrelated to SAEs. There were no significant alterations in mean blood pressure or heart rate observed during the Ang-(1–7) infusion compared to baseline values. Throughout the intervention period, 5 out of 28 (17.8%) patients required vasopressors. Notably, 4 of these patients received low-dose norepinephrine (e.g., < 0.05 mcg/kg min), while one patient necessitated higher doses 3 days after initiating the drug and unfortunately succumbed to septic shock. Importantly, this event was deemed unrelated to drug administration. The clinical course of this patient, the delay from the drug administration, the results of different cultures and the hyperdynamic nature of the shock and with increased SvO2 suggested that the increase in norepinephrine was related to secondary infection and had no time-relationship with the investigational compound. During the study period, another SAE was reported. Shortly after increasing the infusion rate following 24 h of treatment, one patient experienced unexplained sinus bradycardia (e.g., heart rate < 50 beats per min) without concomitant hypotension. This particular patient was concurrently receiving pilocarpine collyrium as a chronic treatment for glaucoma. The attending physician opted to temporarily halt the infusion, as the event was considered potentially related to the study drug. Bradycardia resolved within a few minutes, and the infusion was resumed at a rate of 5 mcg/Kg day without further complications. This event was promptly reported to the Data Safety Monitoring Committee (DSMC) within 6 h, which recommended against increasing the Ang-(1–7) administration in this patient and categorized the bradycardia as possibly related to the study drug. No additional SAEs were observed among the remaining cohort during Phase 1. In Phase 1, secondary infections were observed in 5 (17.8%) patients, and one patient presented clinically significant deep venous thrombosis.

**Fig. 2:**
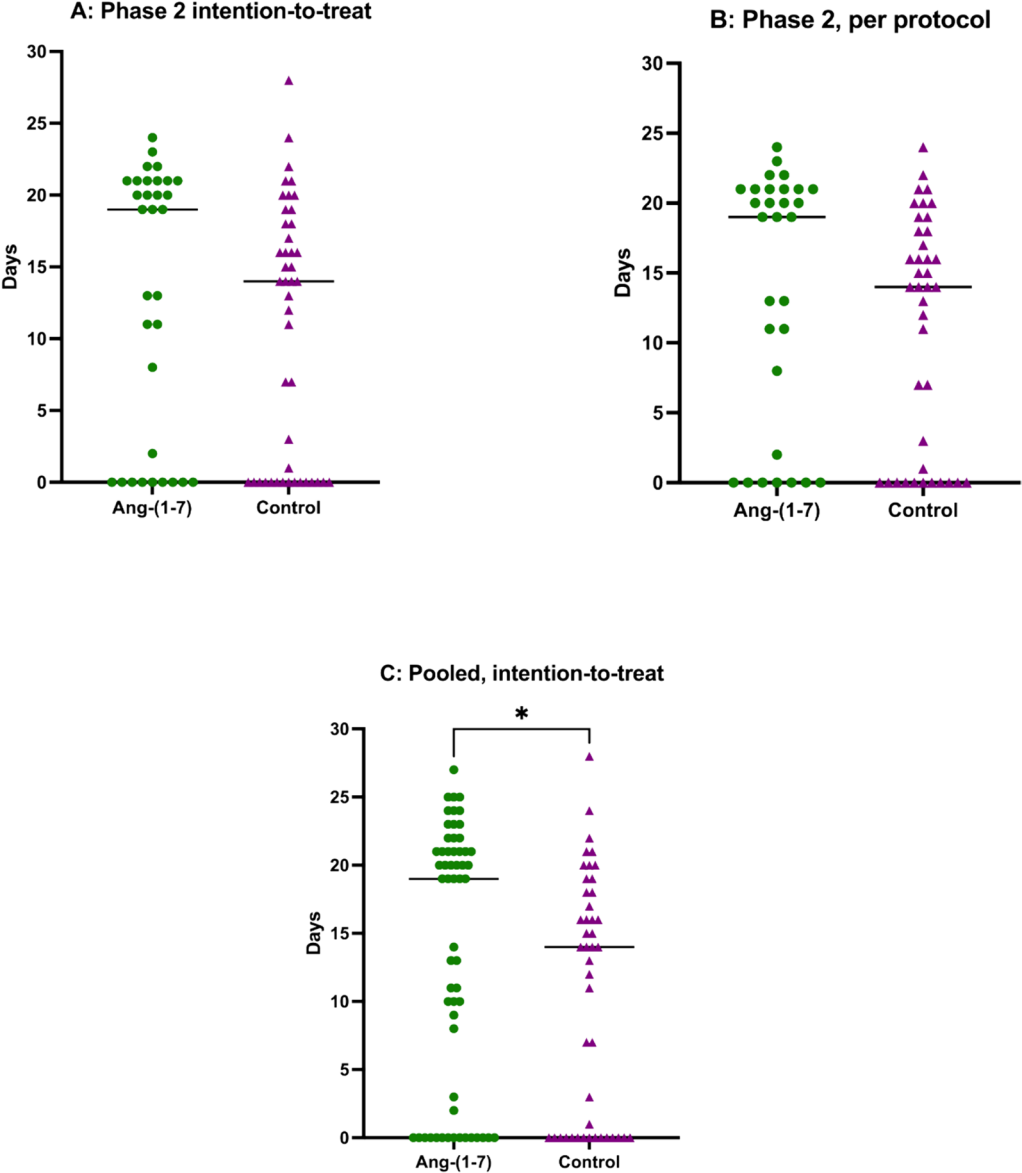
Oxygen free days by day 28. **A** Phase 2, intention-to-treat analysis; **B** Phase 2, per-protocol analysis; **C** pooled analysis Phase 1–2, intention-to-treat

In Phase 2, the median duration of Ang-(1–7) infusion was 4 ± 2 days. Data regarding the primary outcome were available for 77 out of 78 patients (98.7%). One patient in the Ang-(1–7) group was transferred to another healthcare facility after receiving mechanical ventilation for 7 days, and data collection ceased thereafter. There was no significant difference OFDs by day 28 between the two groups, both in the intention-to-treat analysis (19 [0–21] vs. 14 [0–18] days; p = 0.13—Figure 2A) and in the per-protocol analysis (19 [1–21] vs. 14 [0–18] days; p = 0.08—Figure 2B). Results of the primary outcome were consistent in all subgroups (Supplementary Table 1).

When considering all patients from both phases, a total of 103 patients were included in the analysis, primary outcome was available for 58 in the interventional group and 45 in the control group, accounting for 97.1% of patients. In an intention-to-treat analysis, a statistically significant increase in the number of OFDs was observed in the intervention group (19 [0–21] days vs. 14 [0–18] days; absolute difference − 5 days, 95% CI [− 7–0 days]; p = 0.04), as shown in Figure 2C.

#### Secondary outcomes

There was no significant difference in the ICU length of stay among survivors, ICU mortality, ventilator-free days, and requirement of vasopressors between the two groups. In Phase 2, the interventional group had a shorter hospital stay when compared to the control group (Table 2). No significant difference in circulating Ang I, Ang II, Ang-(1–5) and Ang-(1–7) were observed between groups (Supplementary Figure 1).

**Table 2:** Primary and secondary outcomes

Primary outcome	Phase 1	Ang-(1–7)	Control	OR (95%CI)	*p*
OFDs, ITT	19 [4–23]	19 [0–21]	14 [0–18]	–	0.15
OFDs, PP		19 [1.5–21]	14 [0–18]	–	0.08
OFDs, pooled, ITT	19 [0–21]		14 [0–18]	–	0.04
Secondary outcomes					
ICU length of stay	5 [4–7]	6 [5–11]	9 [6–13]	–	0.08
ICU free days by day 28	23 [0–24]	21 [9–23]	18 [7–22]	–	0.5
Hospital length of stay	12 [9–18]	9 [8–14]	14 [10–21]	–	0.01
Ventilator free days by day 28	28 [22–28]	28 [21–28]	28 [11–28]	–	0.3
Need for vasopressors, n (%)	5 (17.8%)	11 (33.3)	19 (42.2)	0.68 (0.25–1.6)	0.6

*OFDs* oxygen free days by day 28, *ITT* intention to treat analysis, *PP* per protocol analysis, *ICU* intensive care unit

**Fig. 3:**
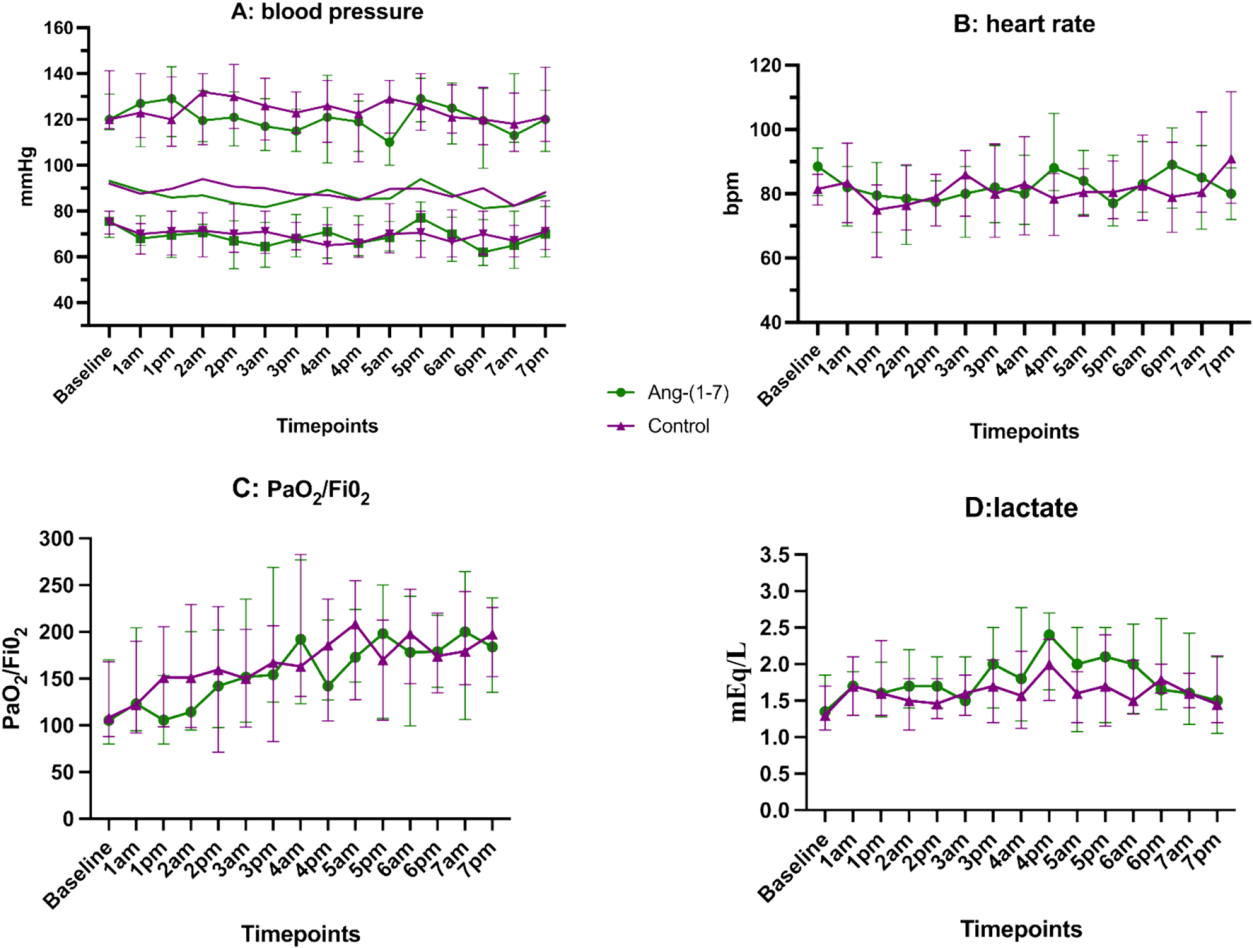
evolution of main physiological variables during Phase 2. **A** systolic, mean, and diastolic arterial pressure; **B** heart rate; **C:**PaO_2_/FiO_2_ and **D** arterial lactate. Lines represent median values and bars interquartile range

No significant difference in biological and physiological variables was observed between groups (Figure 3, Supplemental Figure 2). Reported serious adverse events (SAEs) in the study were, in intervention group: pulmonary embolism and acute cor pulmonale (1 patient), circulatory shock (6 patients), myocardial infarction (1 patient); and placebo group: bilateral pneumothorax (1 patient); circulatory shock (6 patients). All events were immediately evaluated by investigators and clinical committee. None of them was considered related to drug infusion. The pathophysiology of severe COVID-19, onset time of the events and the safety of the drug previously reported in healthy volunteers trials [32, 33] indicate that the SAEs were by causality related to the disease itself or therapeutic interventions other than the drug (i.e.: pneumothorax related to mechanical ventilation barotrauma). Infusion was only discontinued in the presence of significant circulatory shock according to protocol.

## Discussion

The main findings of our study indicate that continuous intravenous infusion of Ang-(1–7) in COVID-19 patients admitted to the ICU was safe. To our knowledge, this is the first human study in which the compound has been administered continuously intravenously for an extended period. Phase 1, focused on safety and dose-finding, was considered a crucial aspect of the study. Previously, there was no consistent data available regarding safe and therapeutic doses of this vasoactive peptide in severe conditions. Although the number of OFDs by day 28 was similar between the two groups in the Phase 2, pooling the results of both phases suggested a potential beneficial effect in treated patients.

These results partially contrast with a randomized clinical trial where Ang-(1–7) infusion did not show improvement in clinically relevant endpoints [28], consistent with earlier findings in a small series of patients [27]. However, notable differences exist between these trials and ours: both previous studies administered a much higher dose of Ang-(1–7) compared to our study (e.g. 0.5 mg/Kg day vs. 10 mcg/Kg day, corresponding to a 50-fold increase in the daily dose). This higher dose could potentially saturate MAS receptors and stimulate AT1 receptors [29], leading to unpredictable biological effects [30]. Additionally, the infusion in those studies was limited to a relatively short 2-h period, possibly limiting the compound biological activity. Another recent placebo-controlled phase 2/3 trial of a MAS receptor activator, BIO101, showed promising results, with a reduced risk of a composite outcome including death or respiratory failure [34]. These findings suggest that non-peptidic ligands may have advantages in overcoming the limitations of Ang-(1–7), due to its rapid metabolism into other peptides and its effect on AT1 receptors in high doses. However, for conditions in which ICU is indicated, intravenous administration of low doses of Ang-(1–7) as in our study might be useful.

We did not observe significant changes in circulating arterial Ang-(1–7) concentrations upon intravenous peptide infusion in our cohort. Several factors may contribute to this observation, including alterations in pulmonary vascular enzymatic activity during COVID-19, trapping of the peptide by MAS receptors or less likely by other angiotensin receptors in the lung circulation, or insufficient passage of the peptide through the pulmonary circulation to modify arterial concentrations. The reversible bradycardia observed in one patient during Phase 1, who was chronically using pilocarpine, suggests that the infused dose was biologically effective. Similarly, there were no significant changes in the arterial concentration of other measured peptides, supporting the possibility of a local action of Ang-(1–7) and the absence of detectable evidence for changes in kidney renin secretion. Reversible bradycardia observed in one patient during Phase 1, who was chronically using pilocarpine, suggests that the infused dose was biologically effective. Similarly, there were no significant changes in the arterial concentration of other measured peptides, supporting the possibility of a local action of Ang-(1–7) and the absence of detectable evidence for a role of an effect of the peptide infusion on the circulating RAS.

Our study has several limitations. Phase 1 was an open-label trial, and caution is needed when interpreting the pooled intention-to-treat analysis. However, given the escalating dose in Phase 1, those patients received a reduced total dose of the investigational compound compared to Phase 2 patients, making it less likely to detect a positive effect of the peptide. Moreover, patient characteristics did not differ significantly at inclusion between the two phases. Phase 2 was terminated prematurely due to low recruitment rates and funding shortages, limiting the interpretation of these data. Additionally, the sample size was determined *a priori* to provide data for a larger clinical trial, and no power calculation was conducted to identify clinically relevant differences between groups. Finally, our study was conducted in two centers in a single city in Brazil, and the generalizability of these findings should be considered carefully. As fewer COVID-19 cases are admitted to the ICU after widespread vaccination, a Phase 3 trial has not been conducted at this time. However, in the event of another COVID-19 wave or any other respiratory syndrome that may benefit from RAS modulation, further investigation in a Phase 3 trial would be warranted to confirm and extend these results.

## Conclusions

The main findings of our study indicate that continuous intravenous infusion of Ang-(1–7) in COVID-19 patients admitted to the ICU was safe. There was no significant change in the primary outcome in per protocol analysis, the We suggest further investigations in this topic considering the exploratory analysis pointing that the intervention could be associated with a reduction of oxygen free days in such patients.

## Supplementary Information

Supplementary Material 1

Supplementary Material 2

## Data Availability

Raw data could be available upon reasonable request to the corresponding author.
