# Supplementary material for: Angiotensin-(1–7) infusion in COVID-19 patients admitted to the ICU: a seamless phase 1–2 randomized clinical trial
Source: Ann Intensive Care. 2024 Sep 4;14:139. doi: 10.1186/s13613-024-01369-0 (PMC11374945; doi:10.1186/s13613-024-01369-0)

Appendix 2: definition of serious adverse event

The following definition of serious adverse event (SAE) was used, according to the Article 2 of Clinical Trials Regulation (EU) No 536/2014: “Any untoward medical occurrence or effect that at any dose requires inpatient hospitalization or prolongation of existing hospitalization, results in persistent or significant disability or incapacity, results in a congenital anomaly or birth defect, is life-threatening or results in death”, considered at the time of the event.
SAEs should include all serious events independent of whether they have a suspected causal relationship to the investigational medicinal product or not.

Supplementary Table 1: Subgroup analysis for oxygen free days by day 28.

**Subgroup (n/n)**		**Ang-(1-7)**	**Controls**	***p***
		**Phase 2**		
**Age**	**< 60 years old** (24/23)	19.5 [3.5-21]	14 [0-18]	0.07
	**≥ 60 years old** (8/22)	9.5 [0-20.5]	13.5 [0-18.25]	0.97
**Sex at birth**	**Male** (21/24)	19 [1-21]	5 [0-16]	0.09
	**Female** (11/18)	19 [0-20]	15 [5.25-19.25]	0.83
**Obesity (BMI > 30 Kg/m)**	**Yes** (16-15)	15 [0-20]	14 [0-16]	0.48
	**No** (18/28)	19 [6.5-21.25]	13 [0-19.75]	0.11
**ACEi/ARBs**	**Yes** (7/10)	11 [0-21]	12.5 [2.25-20.25]	0.78
	**No** (21/29)	19 [5-21]	14 [0-16.5]	0.051
		**Pooled Phase 1+2**		
**Age**	**< 60 years old** (41/23)	20 [9.5-21.5]	14 [0-18]	0.009
	**≥ 60 years old** (19/22)	0 [0-19]	13.5 [0-18.25]	0.52
**Sex at birth**	**Male** (26/24)	19 [6.5-21]	5 [0-16]	0.03
	**Female** (33/18)	14 [0-22]	15 [5.25-19.25]	0.65
**Obesity (BMI > 30 Kg/m)**	**Yes** (26/15)	19 [7.5-21]	14 [0-16]	0.06
	**No** (33/28)	19 [1-22]	13 [0-19.75]	0.19
**ACEi/ARBs**	**Yes** (17/10)	19 [6-22.5]	12.5 [2.25-20.25]	0.33
	**No** (38/29)	19 [0-21]	14 [0-16.5]	0.09

BMI= body mass index; ACEi= angiotensin converting enzyme inhibitors, ARBs= angiotensin receptor blockers.

Supplementary Figure 1: Circulating RAS peptides. A: Ang-(1-7); B: Ang-(1-5); C: Ang-I; D: Ang-II. Points represent median values.

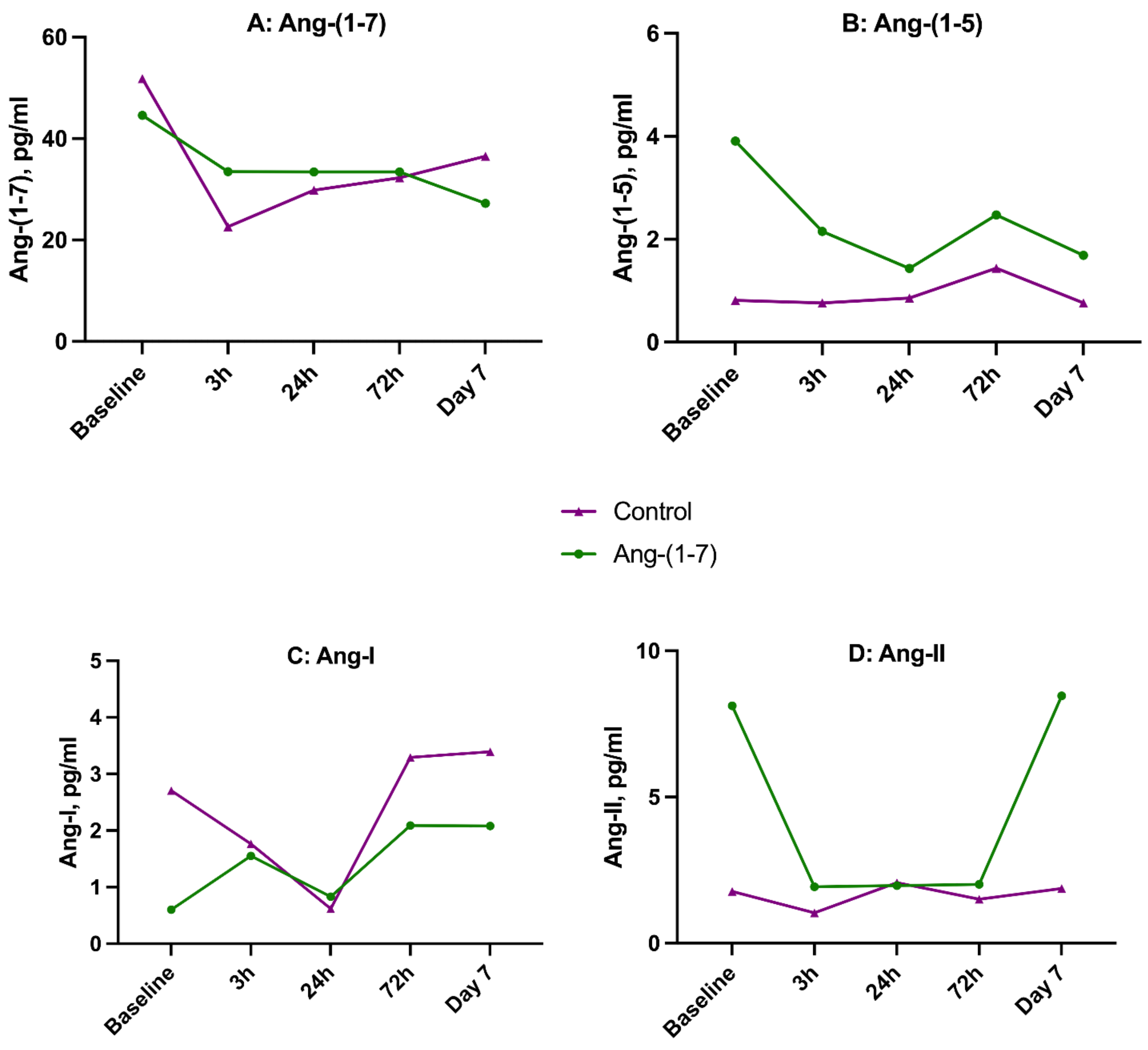

Supplementary Figure 2: time evolution of creatinine (A), C-reactive protein (CRP, B), daily fluid balance (C) and temperature (D). Points represents median values and bars interquartile ranges.

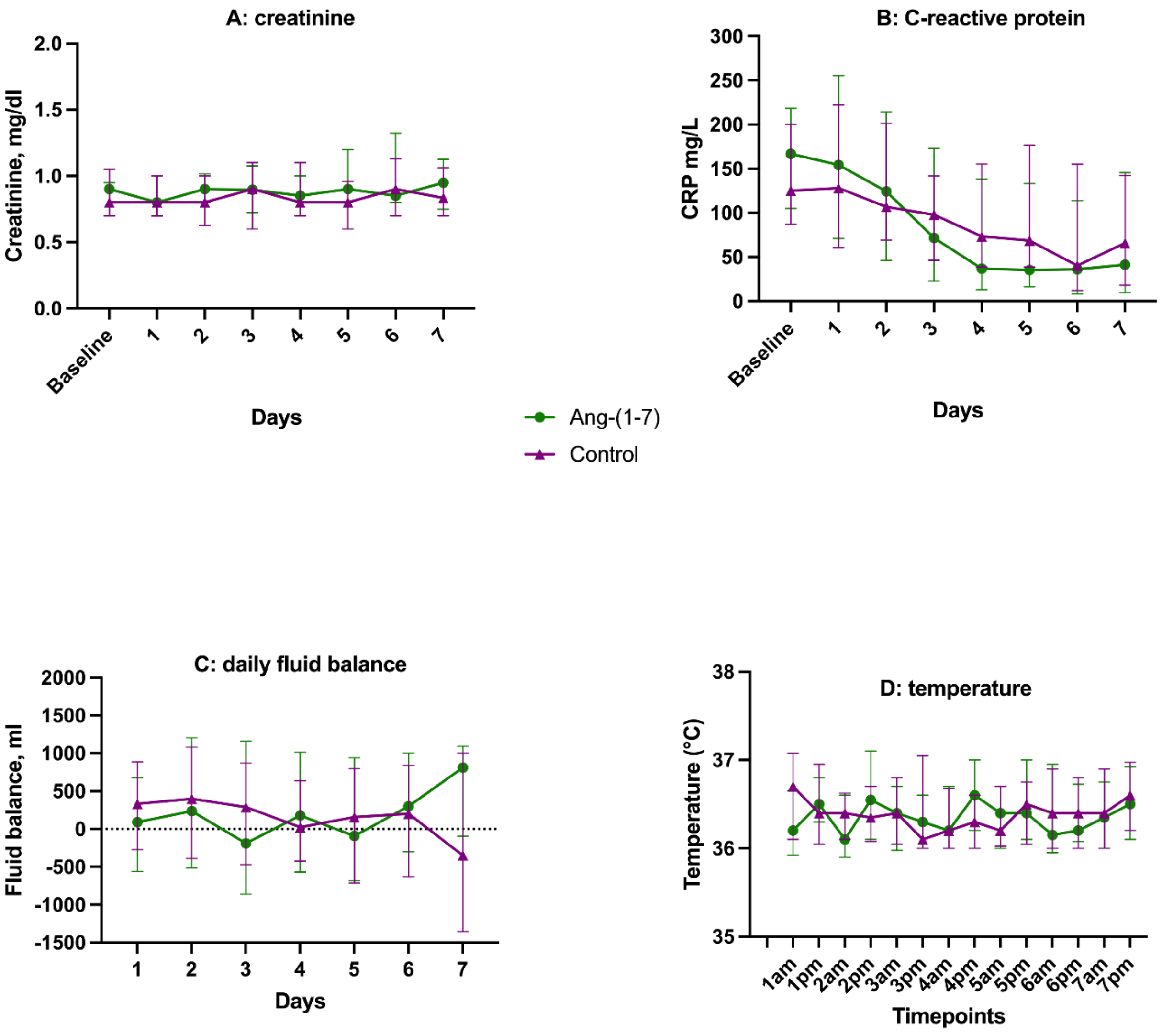

Supplement: Supplementary file 2 — Supplementary Material 2 [file 13613_2024_1369_MOESM2_ESM.pdf]
